# Increased susceptibility of Huh7 cells to HCV replication does not require mutations in RIG-I

**DOI:** 10.1186/1743-422X-7-44

**Published:** 2010-02-19

**Authors:** Dino A Feigelstock, Kathleen B Mihalik, Gerardo Kaplan, Stephen M Feinstone

**Affiliations:** 1 Division of Viral Products, Center for Biologics Evaluation and Research, FDA, 29 Lincoln Drive, Bethesda, MD 20892, USA; 2 Division of Emerging and Transfusion Transmitted Diseases, Center for Biologics Evaluation and Research, FDA, 29 Lincoln Drive, Bethesda, MD 20892, USA

## Abstract

**Background:**

The cytosolic retinoic acid-inducible gene I (RIG-I) is a pattern recognition receptor that senses HCV double-stranded RNA and triggers type I interferon pathways. The clone Huh7.5 of human hepatoma Huh7 cells contains a mutation in RIG-I that is believed to be responsible for the improved replication of HCV in these cells relative to the parental strain. We hypothesized that, in addition to RIG-I, other determinant(s) outside the RIG-I coding sequence are involved in limiting HCV replication in cell culture. To test our hypothesis, we analyzed Huh7 cell clones that support the efficient replication of HCV and analyzed the RIG-I gene.

**Results:**

One clone, termed Huh7D, was more permissive for HCV replication and more efficient for HCV-neomycin and HCV-hygromycin based replicon colony formation than parental Huh7 cells. Nucleotide sequence analysis of the RIG-I mRNA coding region from Huh7D cells showed no mutations relative to Huh7 parental cells.

**Conclusions:**

We derived a new Huh7 cell line, Huh7D, which is more permissive for HCV replication than parental Huh7 cells. The higher permissiveness of Huh7D cells is not due to mutations in the RIG-I protein, indicating that cellular determinants other than the RIG-I amino-acid sequence are responsible for controlling HCV replication. In addition, we have selected Huh7 cells resistant to hygromycin via newly generated HCV-replicons carrying the hygromycin resistant gene. Further studies on Huh7D cells will allow the identification of cellular factors that increased the susceptibility to HCV infection, which could be targeted for anti-HCV therapies.

## Background

Hepatitis C virus (HCV) infects nearly 200 million people worldwide [1]. HCV infection causes chronic liver disease, cirrhosis, and is associated with hepatocellular carcinoma [2]. It is estimated that only 15-40% of infected people resolve acute HCV infection [3], suggesting that host factors are capable of controlling HCV replication in some individuals. However, the host determinants responsible for controlling HCV replication are not well understood. The ability to grow HCV *in vitro *is important for understanding both virologic and immunologic aspects of HCV infections. It has been shown that the JFH1 and the chimeric J6/JFH1 isolate of the 2a genotype of HCV replicate efficiently in Huh7 cells [4,5] and in the highly permissive Huh7.5 and Huh7.5.1 cells derived from the human hepatoma cell line Huh7 [6-9]. Later, production of infectious genotype 1a and 1b viruses [10] was demonstrated in Huh7.5 cells. Further studies showed that the increased permissiveness of Huh7.5 cells results from a mutation (Thr-55-Iso) in the RIG-I gene (retinoic acid-inducible gene I, a DExD/H domain containing RNA helicase, reviewed in [11]) which impairs interferon signaling [12]. In this study, using a technique similar to that used to generate the Huh7.5 cell line, we derived another Huh7 cell line highly permissive for HCV replication that we termed Huh7D. We compared the replication of the genotype 2a J6/JFH1 strain of HCV and the genotype 1b based HCV replicons in Huh7D cells with replication in Huh7 and Huh7.5 cells. We found that while HCV replicated better in Huh7D cells relative to Huh7 cells, no mutations were found in the RIG-I coding region from Huh7D cells, indicating that cellular determinants located outside the RIG-I amino-acid coding sequence are responsible for the higher permissiveness of Huh7D cells for HCV replication.

## Results

### Huh7D cells are more susceptible to HCV replicons than parental Huh7 cells

**Figure 1:**
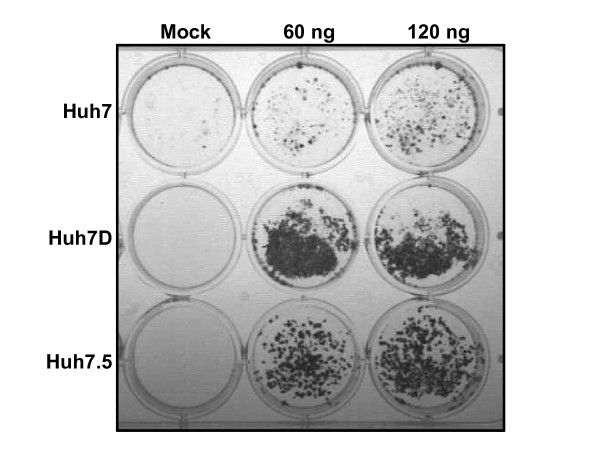
**Transfection of HCV-neo-replicon into Huh7, Huh7D, and Huh7.5 cells**. Coomassie staining of Huh7, Huh7D, and Huh7.5 cells that were transfected with the indicated amounts of HCV-neo replicon and selected for 13 days with G-418 at a concentration of 250 μg/ml.

To compare the susceptibility of cells to HCV replicons, Huh7, Huh7D, and Huh7.5 cells were transfected with HCV-neo-Replicon and selected with 250 μg/ml of G-418. An increased number of neomycin-resistant colonies were observed in Huh7D cells (and control Huh7.5 cells) relative to Huh7 cells (figure 1), irrespective of the amount of transfected RNA. No surviving colonies were observed in replication-defective HCV-neo-Replicon, unrelated RNA (transcribed from pTRI-Xef plasmid from AMBION kit), or no RNA transfected cells (figure 1 and additional file 1). In order to quantify the efficiency of colony formation (ECF), we repeated the experiment using lower amounts of replicon, and obtained ECF of 526, 10,500, and 2631 colonies per μg of transfected RNA for Huh7, Huh7D, and Huh7.5 cells respectively (additional file 1). Wild type HCV-neo replicons obtained by *in vitro *transcription of Sca1 cut plasmid pFK i389neoNS3-3'/WT and other less adapted replicons (Feigelstock et al, unpublished) also yielded more colonies in Huh7D cells relative to Huh7 cells. These results show Huh7D cells have an increased capacity to survive G-418 via HCV-neo-Replicon than parental Huh7 cells, suggesting that the HCV replicon replicates better in Huh7D cells relative to Huh7 cells.

### The increased susceptibility of Huh7D cells to HCV-replicons is independent of the selectable marker coded by the HCV-replicon

**Figure 2:**
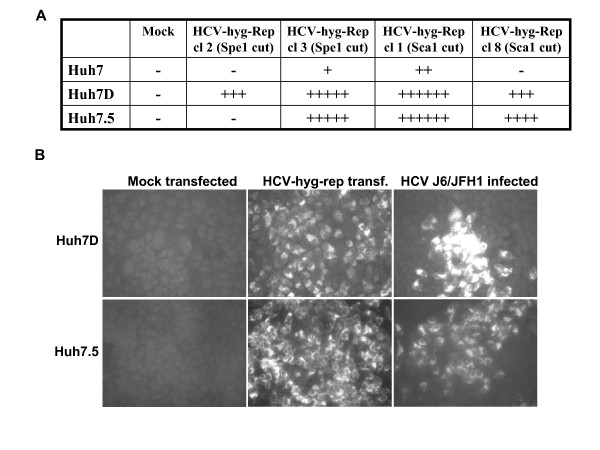
**Transfection of HCV-hyg-replicons into Huh7, Huh7D, and Huh7.5 cells and selection with hygromycin B at a concentration of 65 μg/ml**. A. Relative quantification of surviving colonies. B. Detection of HCV antigens in surviving Huh7D and Huh7.5 cells at 37 days post transfection by immunfluorscence.

To determine whether the selectable marker contained in the HCV-replicon had an effect in the susceptibility of the Huh7 cell clones, we transfected Huh7, Huh7D, and Huh7.5 cells with approximately 100 ng of the indicated HCV-hyg-Replicons and selected cells with 65 μg/ml hygromycin B. At 40 days post-transfection, more hygromycin B resistant colonies were observed in Huh7D and Huh7.5 cells than in Huh7 cells whereas mock-transfected cells did not survive the antibiotic selection (figure 2a). As shown in figure 2a, we were able to select Huh7 colonies resistant to hygromycin B; however, those initially resistant colonies didn't survive longer (more than 60 days) treatment with hygromycin B. Replication of HCV-hyg-Replicon in Huh7D and Huh7.5 cells was confirmed by immunfluorscence analysis (figure 2b). Transfection with HCV-hyg-Replicons yielded a low number of surviving colonies and, given the extended time required for hygromycin to kill Huh7 cells, we needed to make a cell passage resulting in the loss of our ability to accurately quantify the differences in transduction efficiencies. These data indicate that the selectable marker has no effect in the higher susceptibility of the Huh7 clones to HCV replicons.

### Huh7D cells are more susceptible to HCV infection than Huh7 parental cells

**Figure 3:**
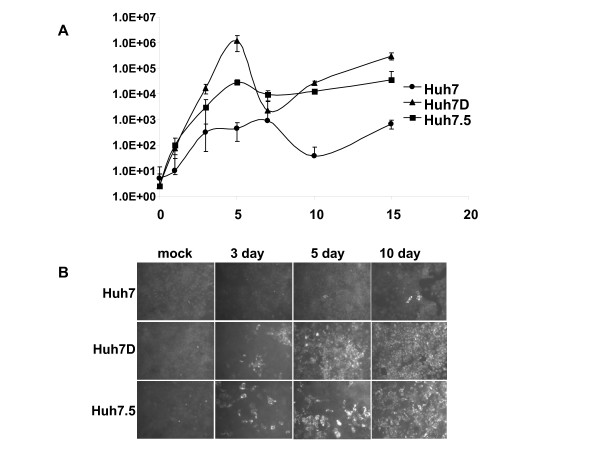
**A. Growth of HCV2a J6/JFH1 in Huh7, Huh7D, and Huh7.5 cells**. The indicated cells were mock infected or infected the J6/JFH1 strain of HCV at an m.o.i. of 0.01. After 6 hours, cells were washed with growth medium three times and passed to 12 well plates. Cells were collected at the indicated time points and frozen at -70°C. Virus was tittered as described in the text. Ffu, focus forming units. Error bars represent the standard error. **B. Growth of HCV2a J6/JFH1 in Huh7, Huh7D, and Huh7.5 cells assessed by IF**. The indicated cells were mock infected or infected the J6/JFH1 strain of HCV at an m.o.i. of 0.01. After 6 hours, cells were washed with growth medium for three times and passed to 96 well plates. HCV antigen was detected at the indicated time points by immunfluorscence.

We next wanted to determine whether Huh7D cells were more susceptible to HCV replication than parental Huh7 cells when using the J6/JFH1 infectious clone. To do so, we infected Huh7, Huh7D, and Huh7.5 cells with HCV-J6/JFH1 at an m.o.i. of 0.01 and analyzed virus growth at 0, 1, 3, 5, 7, 10, and 15 dpi using an IF end-point dilution titration assay and by IF on infected cells. HCV J6/JFH1 grew faster in Huh7D and Hu7.5 cells relative to Huh7 cells (figure 3a), which is consistent with our results showing that the Huh7D and Huh7.5 cells were more susceptible to HCV replicons than the parental Huh7 cells. Cells passed to 96 well plates were stained with anti-HCV antibodies at 3, 5, and 10 dpi, and HCV antigen was detected by IF analysis (figure 3b). In agreement with the titration data, Huh7D and Huh7.5 cells showed an increase in the percentage of infected cells relative to Huh7 cells. These results show that J6/JFH1 virus grew better in Huh7D cells and in Huh7.5 cells than in Huh7 cells. In order to discard the possibility that the J6/JFH1 virus grew better in Huh7D cells relative to Huh7 and Huh7.5 cells because it was produced in Huh7D cells (and therefore may have acquired Huh7D adaptive mutations), we sequenced the full length genome of the J6/JFH1 virus we used to inoculate the cells. We found no differences in the nucleotide sequence with respect to the J6/JFH1 sequence present in the plasmid, except in three positions that showed a mixture of two nucleotides (T2667T/C; A7150G/A; and T7667T/A). In addition, we repeated the experiment shown in figure 3 but using a J6/JFH1 virus that had been grown only in Huh7.5 cells and therefore there was no chance that the virus had adapted to the Huh7D cells prior to studying the replication of the virus in those cells. Again we saw higher titers in Huh7D relative to Huh7 cells (2 logs). This result suggest that the observed higher susceptibility of Huh7D cells to J6/JFH1 infection is not due to adaptation of the virus to Huh7D cells. Furthermore, JFH1 virus (also not passaged in Huh 7D cells) also grew better in Huh7D and Huh7.5 cells relative to Huh7 cells (not shown).

### There are no amino-acid substitutions in the RIG-I coding region from Huh7D cells

In order to determine if the increased susceptibility of Huh7D cells to HCV replication was due to mutations in RIG-I as observed in Huh7.5 cells [12], we sequenced the full coding region of the RIG-I mRNA from Huh7, Huh7D, and Huh7.5 cells by RT-PCR. The RIG-I coding region sequence was identical in Huh7D and parental Huh7 cells while the expected ACA to ATA (Thr-55-Iso) substitution was found in Huh7.5 cells (additional file 2).

## Discussion

In this study we derived Huh7D cells, a single cell clone of replicon-cured Huh7 cells. We show that as the previously reported Huh7.5 cells, Huh7D cells are more permissive to HCV replication than parental Huh7 cells. Huh7D cells were similarly permissive to HCV replicon (neo and hyg) replication as Huh7.5 cells, and were at least as permissive to HCV J6/JFH1 infection as Huh7.5 cells. Sequencing of the coding region of RIG-I mRNA from Huh7D cells, as opposed to the RIG-I coding region from Huh7.5 cells, showed no mutations when compared to the RIG-I coding region from parental Huh7 cells. This indicates that mutations in RIG-I coding region are not responsible for the higher permissiveness of Huh7D cells to HCV replication. This is in agreement with recent observations indicating that an intact RIG-I signaling pathway does not necessarily limit HCV replication in Huh-7 cells [13].

At this time we have not identified the factor/s responsible for the higher permissiveness of Huh7D cells. Other than RIG-I cellular factors affecting HCV replication have been identified. Reconstituted Toll like receptor 3 (TLR3) in Huh7 and Huh7.5 cells senses HCV infection independently of RIG-I, and triggers an antiviral state [14]. Class III Phosphatidylinositol 4-Kinase alpha and beta were recently identified as regulators of hepatitis C virus replication in Huh7 cells [15]. A screening using siRNA identified host genes that modulate HCV replication, including host genes related to the RNAi pathway [16], transcription factors, transporter proteins, and others [17].

In order to obtain a Huh7 cell line with even higher permissiveness for HCV replication, we selected double cured Huh7 cells (cells selected with HCV-neo replicon, cured, selected with HCV-hyg replicon, and cured again, or cells selected twice with HCV-neo replicons), but we couldn't obtain Huh7 cells with higher permissiveness for HCV replicon replication or HCV infection (not shown). The failure to obtain cells that are more permissive to HCV replication by successive curing of transfected cells suggests that cellular mechanisms involved in HCV replication are difficult to alter. It is also possible that interferon signaling is the major cellular mechanism for controlling HCV replication (and/or the easiest to alter), and once this pathway is altered, few other (alterable) pathways are left to facilitate HCV replication.

We have shown that Huh7D cells are more permissive than Huh7 cells not only for a replicon with the neo selectable marker, but also for an HCV replicon which expresses the hygromycin resistance gene. We were able to select Huh7D (and Huh7.5) but not Huh7 cells resistant to hygromycin B after HCV-hyg replicon transfection. Although we were able to initially select Huh7 cells resistant to hygromycin B, treatment with the antibiotic for periods longer than 60 days induced the extinction of the Huh7 colonies. This observation suggest that replication of HCV-hyg-rep in Huh7 cells is limited, but the lesser susceptibility of Huh7 cells to hygromycin permits longer surviving of the colonies. In fact, while mock transfected Huh7 cells survive 250 μg/ml Neomycin for about 2 weeks, mock transfected Huh7 cells survive 65 μg/ml hygromycin for about 30 days. Obtaining resistance to hygromycin B was more difficult and less efficient than obtaining resistance to neomycin. We don't know the reason underlying this observation, which together with the fact that resistance of Huh7 cells to hygromycin B via HCV-hyg replicons was not widely reported, suggest the presence of intrinsic barriers for Huh7 cells to survive hygromycin B using HCV-hyg replicons.

## Conclusion

In this study we derived a new Huh7 cell line (Huh7D) which is more permissive for HCV replication than parental Huh7 cells. The permissiveness of Huh7D cells is not due to mutations in the RIG-I protein, as reported for the widely used Huh7.5 cells. More experiments are needed to elucidate if the cellular determinant/s responsible for the higher permissiveness of Huh7D cells are related to the interferon or other cellular pathways.

## Methods

### Cells

Huh7 and Huh7.5 cells were a gift from Jake Liang. Huh7, Huh7D, and Huh7.5 cells were grown in DMEM (Gibco) supplemented with 10% bovine calf serum (Atlanta Biologicals), L-glutamine (Gibco), penicillin and streptomycin (Gibco).

### Viruses

The JFH1 virus was a gift from Takaji Wakita. J6/JFH1 virus was obtained by transfection of Huh7D cells (see below) with *in vitro *transcribed HCV J6/JFH1 RNA. HCV J6/JFH1 RNA was obtained from plasmid pFL- J6/JFH1 (a gift from Charles Rice) that was cut with Xba1 and transcribed with T7 RNA polymerase (T7 Megascript AMBION).

### Generation of HCV replicon containing the neomycin resistance gene ("HCV-neo-Rep")

HCV-neo-Replicon and replication-defective HCV replicon were obtained as previously described [18]. Briefly, plasmid pFK i389neoNS3-3'/NK5.1 coding for a highly permissive HCV-neo replicon harboring several replication enhancing mutations [19] and plasmid pFK i389neoNS3-3'/delta5B [18] (kindly provided by Ralph Bartenschlager) were cut with restriction enzyme Sca1, and *in vitro *transcribed using T7 Megascript kit (AMBION).

### Generation of HCV replicon containing the hygromycin resistance gene ("HCV-hyg-Rep")

To obtain HCV replicons carrying the hygromycin resistance gene ("HCV-hyg-Rep") we replaced the neomycin resistance gene with the hygromycin resistance gene in plasmid pFK i389neoNS3-3'/NK5.1 using restriction enzymes Asc1 and Pme1. Hygromycin resistance gene was obtained by PCR using plasmid pIREShyg (Clontech) as template and sense oligo AACTAAAGGCGCGCCATGGATAGATCCGGAAAGCCTGAACTCAC (carrying the Asc1 restriction site) and anti-sense oligo AGTTATGGTTTAAACCTATTCCTTTGCCCTCGGACGAGTGCTGGG or anti-sense oligo AGTTATGGTTTAAACCTATTCCTTTGCCCTCGGACGAGTGCTGGGGCGTCGGTTTCCACTATCGGCGAGAACTTCTAC (both carrying the Pme1 restriction site). The later anti-sense oligo is designed to mutate the Sca1 restriction site present at the 3' end of the hygromycin resistant gene, without changing the coded amino-acid (restriction enzyme Sca1 is used to linearize the vector in order to make *in vitro *transcripts, see below). The PCR products were cut with restriction enzymes Asc1 and Pme1 and ligated to plasmid pFK i389neoNS3-3'/NK5.1 that was cut with same restriction enzymes to obtain pFK i389hygNS3-3'/NK5.1 and pFK i389hygscalessNS3-3'/NK5.1. The resultant recombinant plasmids were transformed into TOP10 competent bacteria (Invitrogen). Bacteria clones carrying the HCV-hyg-Replicons were confirmed by restriction analysis and sequencing. Plasmids pFK i389hygNS3-3'/NK5.1 (clones 2 and 3) were cut with restriction enzyme Spe 1 (generating a replicon with additional 4 nucleotides at the 3' end) and plasmids pFK i389hygscalessNS3-3'/NK5.1 (clones 1 and 8) were cut with Sca1 (to obtain a replicon with the authentic 3' end sequence), and *in vitro *transcribed using T7 Megascript kit (AMBION).

### Generation of Huh7D cells

Huh7 cells grown in 12 well plates were transfected with approximately 100 or 200 ng of HCV-neo-replicon using as a facilitator 3 μl of lipofectamine (Invitrogen) in 200 μl of Optimem (Gibco). At 5 hours post transfection, medium was replaced with DMEM containing 10% fetal calf serum and antibiotics. Replicon harboring cells were selected with G-418 (Roche) at a concentration of 250 μg/ml for 20 days. Single cell clones obtained by end-point dilution were grown and tested by PCR and Southern blot for the (lack of) incorporation of the Neomycin resistance gene into the genome and by Northern blot for the presence of the RNA transcript corresponding to the replicon (not shown). Expression of HCV protein was assessed by immunfluorscence using an anti-NS5a antibody (not shown). Clone D, which had high levels of HCV protein, harbored the HCV replicon, and did not have the Neo gene integrated into the cellular genome, was selected for further analysis. Clone D was "cured" from the replicon using a strategy similar as the one previously described [6]. Briefly, Clone D cells were passed four times at 7 or 8 day interval in absence of G-418 and treated with human Interferon (Sigma I2396) at a concentration of 100 IU/ml. After two weeks, cells were tested for the absence of the HCV replicon by RT-PCR and their susceptibility to G-418. The expected PCR band was not detected, and cells regained susceptibility to G-418 at a concentration of 250 μg/ml, which indicated that the Clone D cells were cured from the HCV replicon, and were named Huh7D cells.

### Transfection of Huh7 cells with HCV replicons

Huh7, Huh7D, and Huh7.5 cells grown in 12 well plates were transfected with different amounts of HCV-neo- or HCV-hyg replicons using as a facilitator 3 μl of lipofectamine (Invitrogen) in 200 μl of Optimem (Gibco). At 5 hours post transfection, medium was replaced with DMEM containing 10% fetal calf serum and antibiotics. At 24 hours post transfection, medium was replaced with same medium containing G-418 (Roche) at a concentration of 250 μg/ml (for HCV-neo-replicon transfected cells) or hygromycin B (Roche) at a concentration of 65 μg/ml (for HCV-hyg-replicon transfected cells). To measure susceptibility to HCV replication, the HCV-neo-replicon transfected cells were fixed 13 or 15 days post transfection and stained with a solution of 50% methanol and 10% acetic acid containing 0.6 g/L of Comassie brilliant blue. The HCV-hyg-replicon transfected cells were split in medium containing 65 μg/ml hygromycin B, and colonies were stained with anti-HCV specific antibody as described below.

### Detection of HCV antigen by immunfluorscence (IF)

Cells transfected with HCV-hyg-replicon or infected with HCV J6/JFH1 were fixed with methanol, blocked with a solution containing 1% BSA and 0.2% non-fat milk in 1 × PBS, treated with a 1:200 dilution in 0.05% tween 20 in 1 × PBS of a serum from a persistently infected chimpanzee that carried high levels of anti-HCV antibodies [20] for 2 hours, washed with 1 × PBS, stained with FITC-conjugated goat anti-human antibody (KPL), washed, and observed in the microscope.

### Infection of Huh7 cells with HCV J6/JFH1 and titration of progeny virus

Huh7, Huh7D, and Huh7.5 cells grown in 6-well plates were mock infected or infected with HCV-J6/JFH1 at an moi of 0.01. At 6 hours post infection, cells were washed three times with DMEM containing 10% FCS and split into 12-well plates (for titration of total progeny virus) and 96-well plates (for IF analysis, described above). At 0, 1, 3, 5, 7, and 10 dpi, the 12-well plates were frozen at -70°C. For later time points, cells in one 12-well plate were split and treated as described above. Total virus from each time point was recovered by freezing and thawing the cells 3 times. Viral titers were obtained in Huh7.5 cells infected with 10-fold serial dilutions of the cell extracts followed by detection of viral antigens by IF analysis at three days post-infection as described above.

### Amplification and sequencing of RIG-I mRNA

Total RNA was extracted from Huh7, Huh7D, and Huh7.5 cells grown in T25 flasks using Trizol reagent as recommended by the manufacturer (Invitrogen). cDNA was synthesized using 4 μg of each RNA, SuperScript III reverse transcriptase (Invitrogen), and random primers. PCR amplification of RIG-I transcripts was performed using RIG-I specific primers RIG-I 91+ (5'-CTACCCGGCTTTAAAGCTAG-3 and RIG-I 3020- (5'-CGATCCATGATTATACCCAC-3'). Nested-PCR was performed using RIG-I-specific primers RIG-I 121+ (5'-CCTGCGGGGAACGTAGCTAG-3') and RIG-I 530- (5'-AATGATATCGGTTGGGATAA-3'), RIG-I 421+ (5'-CCATTGAAAGTTGGGATTTC-3') and RIG-I 1410- (5'-TGGCATCCCCAACACCAACC-3'), RIG-I 421+ and RIG-I 2990-(5'-TCTTCTCCACTCAAAGTTAC-3'). The Expand High Fidelity system (Roche) was used for PCR amplifications as described by the manufacturer. PCR products were run in agarose gels and purified using gene-elute agarose gel columns (Sigma) and sequenced (ABI-prism) using the above mentioned oligos and oligos RIG-I 474- (5'-GTAATCTATACTCCTCCAAC-3'), RIG-I 1331- (5'-AGATCAGAAACTTGGAGGAT-3'), RIG-I 2281+ (5'-AGTGCAATCTTGTCATCCTT-3'), and RIG-I 2360- (5'-TCTTGCTCTTCCTCTGCCTC-3').

## Competing interests

The authors declare that they have no competing interests.

## Authors' contributions

DF, GK, and SF conceived the study, its design, and coordination. DF isolated the Huh7D cells, characterized them, and generated the newly reported HCV-hyg replicons. DF performed the transfections and immunoassays. DF and KM performed the growth curves for the virus in the three cell lines. DF drafted the manuscript with the help of SF, GK, and KM. All authors approved the final version.

## Supplementary Material

Additional file 1 **Transfection of HCV-neo-replicon into Huh7, Huh7D, and Huh7.5 cells**. Coomassie staining of Huh7, Huh7D, and Huh7.5 cells that were mock transfected or transfected with a replication-defective HCV replicon, unrelated RNA, or with the indicated amounts of HCV-neo-replicon, and selected for 15 days with G-418 at a concentration of 250 μg/ml. Click here for file

Additional file 2 **Alignment of nucleotide sequences of RIG-I mRNA from Huh7, Huh7D, and Huh7.5 cells**. Total RNA was extracted from Huh7, Huh7D, and Huh7.5 cells and reverse transcribed using random primers. RIG-I mRNA was amplified by PCR using RIG-I specific primers as indicated in the Materials and Methods section. The alignment of the three sequences was performed using the Clustal method. Click here for file
