# Supplementary figures and images for: Increased susceptibility of Huh7 cells to HCV replication does not require mutations in RIG-I
Source: Virol J. 2010 Feb 19;7:44. doi: 10.1186/1743-422X-7-44 (PMC2831881; doi:10.1186/1743-422X-7-44)

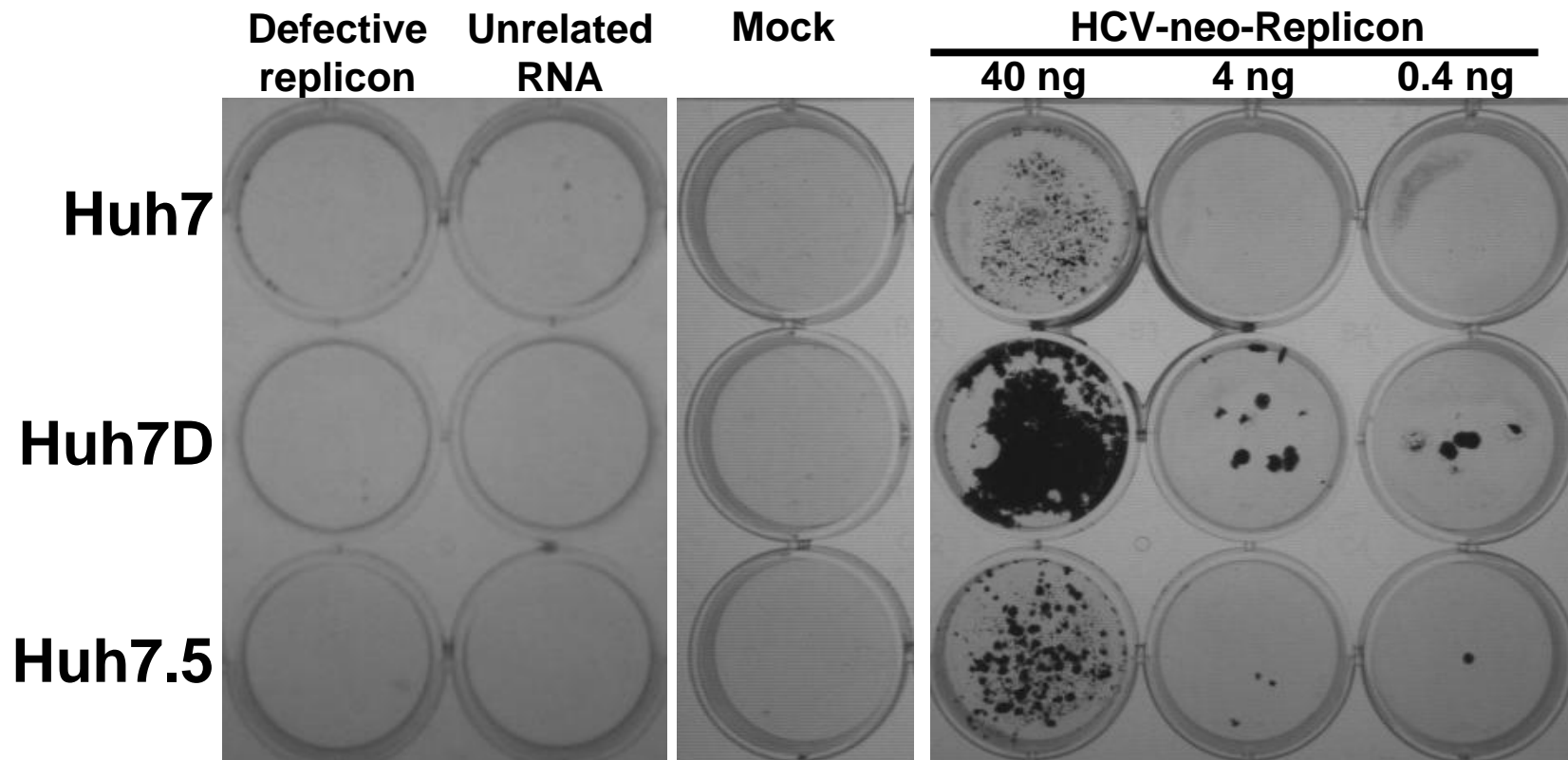

Supplement: Additional file 1 — Transfection of HCV-neo-replicon into Huh7, Huh7D, and Huh7.5 cells. Coomassie staining of Huh7, Huh7D, and Huh7.5 cells that were mock transfected or transfected with a replication-defective HCV replicon, unrelated RNA, or with the indicated amounts of HCV-neo-replicon, and selected for 15 days with G-418 at a concentration of 250 μg/ml. [file 1743-422X-7-44-S1.PDF]
