# Supplementary material for: Increased susceptibility of Huh7 cells to HCV replication does not require mutations in RIG-I
Source: Virol J. 2010 Feb 19;7:44. doi: 10.1186/1743-422X-7-44 (PMC2831881; doi:10.1186/1743-422X-7-44)
Supplement: Additional file 2 — Alignment of nucleotide sequences of RIG-I mRNA from Huh7, Huh7D, and Huh7.5 cells. Total RNA was extracted from Huh7, Huh7D, and Huh7.5 cells and reverse transcribed using random primers. RIG-I mRNA was amplified by PCR using RIG-I specific primers as indicated in the Materials and Methods section. The alignment of the three sequences was performed using the Clustal method. [file 1743-422X-7-44-S2.PDF]

```
Huh7      ATGACCACCGAGCAGCGACGCAGCCTGCAAGCCTTCCAGGATTATATCCGGAAGACCCTG
Huh7D     ATGACCACCGAGCAGCGACGCAGCCTGCAAGCCTTCCAGGATTATATCCGGAAGACCCTG
Huh7.5    ATGACCACCGAGCAGCGACGCAGCCTGCAAGCCTTCCAGGATTATATCCGGAAGACCCTG
          1........10........20........30........40........50.........
Huh7      GACCCTACCTACATCCTGAGCTACATGGCCCCCTGGTTTAGGGAGGAAGAGGTGCAGTAT
Huh7D     GACCCTACCTACATCCTGAGCTACATGGCCCCCTGGTTTAGGGAGGAAGAGGTGCAGTAT
Huh7.5    GACCCTACCTACATCCTGAGCTACATGGCCCCCTGGTTTAGGGAGGAAGAGGTGCAGTAT
          61.......70........80........90........100.......110........
Huh7      ATTCAGGCTGAGAAAAACAACAAGGGCCCAATGGAGGCTGCCACACTTTTTCTCAAGTTC
Huh7D     ATTCAGGCTGAGAAAAACAACAAGGGCCCAATGGAGGCTGCCACACTTTTTCTCAAGTTC
Huh7.5    ATTCAGGCTGAGAAAAACAACAAGGGCCCAATGGAGGCTGCCATACTTTTTCTCAAGTTC
          121......130.......140.......150.......160.......170........
Huh7      CTGTTGGAGCTCCAGGAGGAAGGCTGGTTCCGTGGCTTTTTGGATGCCCTAGACCATGCA
Huh7D     CTGTTGGAGCTCCAGGAGGAAGGCTGGTTCCGTGGCTTTTTGGATGCCCTAGACCATGCA
Huh7.5    CTGTTGGAGCTCCAGGAGGAAGGCTGGTTCCGTGGCTTTTTGGATGCCCTAGACCATGCA
          181......190.......200.......210.......220.......230........
Huh7      GGTTATTCTGGACTTTATGAAGCCATTGAAAGTTGGGATTTCAAAAAAATTGAAAAGTTG
Huh7D     GGTTATTCTGGACTTTATGAAGCCATTGAAAGTTGGGATTTCAAAAAAATTGAAAAGTTG
Huh7.5    GGTTATTCTGGACTTTATGAAGCCATTGAAAGTTGGGATTTCAAAAAAATTGAAAAGTTG
          241......250.......260.......270.......280.......290........
Huh7      GAGGAGTATAGATTACTTTTAAAACGTTTACAACCAGAATTTAAAACCAGAATTATCCCA
Huh7D     GAGGAGTATAGATTACTTTTAAAACGTTTACAACCAGAATTTAAAACCAGAATTATCCCA
Huh7.5    GAGGAGTATAGATTACTTTTAAAACGTTTACAACCAGAATTTAAAACCAGAATTATCCCA
          301......310.......320.......330.......340.......350........
Huh7      ACCGATATCATTTCTGATCTGTCTGAATGTTTAATTAATCAGGAATGTGAAGAAATTCTA
Huh7D     ACCGATATCATTTCTGATCTGTCTGAATGTTTAATTAATCAGGAATGTGAAGAAATTCTA
Huh7.5    ACCGATATCATTTCTGATCTGTCTGAATGTTTAATTAATCAGGAATGTGAAGAAATTCTA
          361......370.......380.......390.......400.......410........
Huh7      CAGATTTGCTCTACTAAGGGGATGATGGCAGGTGCAGAGAAATTGGTGGAATGCCTTCTC
Huh7D     CAGATTTGCTCTACTAAGGGGATGATGGCAGGTGCAGAGAAATTGGTGGAATGCCTTCTC
Huh7.5    CAGATTTGCTCTACTAAGGGGATGATGGCAGGTGCAGAGAAATTGGTGGAATGCCTTCTC
          421......430.......440.......450.......460.......470........
Huh7      AGATCAGACAAGGAAAACTGGCCCAAAACTTTGAAACTTGCTTTGGAGAAAGAAAGGAAC
Huh7D     AGATCAGACAAGGAAAACTGGCCCAAAACTTTGAAACTTGCTTTGGAGAAAGAAAGGAAC
Huh7.5    AGATCAGACAAGGAAAACTGGCCCAAAACTTTGAAACTTGCTTTGGAGAAAGAAAGGAAC
          481......490.......500.......510.......520.......530........
Huh7      AAGTTCAGTGAACTGTGGATTGTAGAGAAAGGTATAAAAGATGTTGAAACAGAAGATCTT
Huh7D     AAGTTCAGTGAACTGTGGATTGTAGAGAAAGGTATAAAAGATGTTGAAACAGAAGATCTT
Huh7.5    AAGTTCAGTGAACTGTGGATTGTAGAGAAAGGTATAAAAGATGTTGAAACAGAAGATCTT
          541......550.......560.......570.......580.......590........
Huh7      GAGGATAAGATGGAAACTTCTGACATACAGATTTTCTACCAAGAAGATCCAGAATGCCAG
Huh7D     GAGGATAAGATGGAAACTTCTGACATACAGATTTTCTACCAAGAAGATCCAGAATGCCAG
Huh7.5    GAGGATAAGATGGAAACTTCTGACATACAGATTTTCTACCAAGAAGATCCAGAATGCCAG
          601......610.......620.......630.......640.......650........
Huh7      AATCTTAGTGAGAATTCATGTCCACCTTCAGAAGTGTCTGATACAAACTTGTACAGCCCA
Huh7D     AATCTTAGTGAGAATTCATGTCCACCTTCAGAAGTGTCTGATACAAACTTGTACAGCCCA
Huh7.5    AATCTTAGTGAGAATTCATGTCCACCTTCAGAAGTGTCTGATACAAACTTGTACAGCCCA
          661......670.......680.......690.......700.......710........
Huh7      TTTAAACCAAGAAATTACCAATTAGAGCTTGCTTTGCCTGCTATGAAAGGAAAAAACACA
Huh7D     TTTAAACCAAGAAATTACCAATTAGAGCTTGCTTTGCCTGCTATGAAAGGAAAAAACACA
Huh7.5    TTTAAACCAAGAAATTACCAATTAGAGCTTGCTTTGCCTGCTATGAAAGGAAAAAACACA
          721......730.......740.......750.......760.......770........
Huh7      ATAATATGTGCTCCTACAGGTTGTGGAAAAACCTTTGTTTCACTGCTTATATGTGAACAT
Huh7D     ATAATATGTGCTCCTACAGGTTGTGGAAAAACCTTTGTTTCACTGCTTATATGTGAACAT
Huh7.5    ATAATATGTGCTCCTACAGGTTGTGGAAAAACCTTTGTTTCACTGCTTATATGTGAACAT
          781......790.......800.......810.......820.......830........
```

```
Huh7          CATCTTAAAAAATTCCCACAAGGACAAAAGGGGAAAGTTGTCTTTTTTGCGAATCAGATC
Huh7D         CATCTTAAAAAATTCCCACAAGGACAAAAGGGGAAAGTTGTCTTTTTTGCGAATCAGATC
Huh7.5        CATCTTAAAAAATTCCCACAAGGACAAAAGGGGAAAGTTGTCTTTTTTGCGAATCAGATC
              841......850.......860.......870.......880.......890........
Huh7          CCAGTGTATGAACAGCAGAAATCTGTATTCTCAAAATACTTTGAAAGACATGGGTATAGA
Huh7D         CCAGTGTATGAACAGCAGAAATCTGTATTCTCAAAATACTTTGAAAGACATGGGTATAGA
Huh7.5        CCAGTGTATGAACAGCAGAAATCTGTATTCTCAAAATACTTTGAAAGACATGGGTATAGA
              901......910.......920.......930.......940.......950........
Huh7          GTTACAGGCATTTCTGGAGCAACAGCTGAGAATGTCCCAGTGGAACAGATTGTTGAGAAC
Huh7D         GTTACAGGCATTTCTGGAGCAACAGCTGAGAATGTCCCAGTGGAACAGATTGTTGAGAAC
Huh7.5        GTTACAGGCATTTCTGGAGCAACAGCTGAGAATGTCCCAGTGGAACAGATTGTTGAGAAC
              961......970.......980.......990.......1000......1010.......
Huh7          AATGACATCATCATTTTAACTCCACAGATTCTTGTGAACAACCTTAAAAAGGGAACGATT
Huh7D         AATGACATCATCATTTTAACTCCACAGATTCTTGTGAACAACCTTAAAAAGGGAACGATT
Huh7.5        AATGACATCATCATTTTAACTCCACAGATTCTTGTGAACAACCTTAAAAAGGGAACGATT
              1021.....1030......1040......1050......1060......1070.......
Huh7          CCATCACTATCCATCTTTACTTTGATGATATTTGATGAATGCCACAACACTAGTAAACAA
Huh7D         CCATCACTATCCATCTTTACTTTGATGATATTTGATGAATGCCACAACACTAGTAAACAA
Huh7.5        CCATCACTATCCATCTTTACTTTGATGATATTTGATGAATGCCACAACACTAGTAAACAA
              1081.....1090......1100......1110......1120......1130.......
Huh7          CACCCGTACAATATGATCATGTTTAATTATCTAGATCAGAAACTTGGAGGATCTTCAGGC
Huh7D         CACCCGTACAATATGATCATGTTTAATTATCTAGATCAGAAACTTGGAGGATCTTCAGGC
Huh7.5        CACCCGTACAATATGATCATGTTTAATTATCTAGATCAGAAACTTGGAGGATCTTCAGGC
              1141.....1150......1160......1170......1180......1190.......
Huh7          CCACTGCCCCAGGTCATTGGGCTGACTGCCTCGGTTGGTGTTGGGGATGCCAAAAACACA
Huh7D         CCACTGCCCCAGGTCATTGGGCTGACTGCCTCGGTTGGTGTTGGGGATGCCAAAAACACA
Huh7.5        CCACTGCCCCAGGTCATTGGGCTGACTGCCTCGGTTGGTGTTGGGGATGCCAAAAACACA
              1201.....1210......1220......1230......1240......1250.......

Huh7          GATGAAGCCTTGGATTATATCTGCAAGCTGTGTGCTTCTCTTGATGCGTCAGTGATAGCA
Huh7D         GATGAAGCCTTGGATTATATCTGCAAGCTGTGTGCTTCTCTTGATGCGTCAGTGATAGCA
Huh7.5        GATGAAGCCTTGGATTATATCTGCAAGCTGTGTGCTTCTCTTGATGCGTCAGTGATAGCA
              1261.....1270......1280......1290......1300......1310.......
Huh7          ACAGTCAAACACAATCTGGAGGAACTGGAGCAAGTTGTTTATAAGCCCCAGAAGTTTTTC
Huh7D         ACAGTCAAACACAATCTGGAGGAACTGGAGCAAGTTGTTTATAAGCCCCAGAAGTTTTTC
Huh7.5        ACAGTCAAACACAATCTGGAGGAACTGGAGCAAGTTGTTTATAAGCCCCAGAAGTTTTTC
              1321.....1330......1340......1350......1360......1370.......
Huh7          AGGAAAGTGGAATCACGGATTAGCGACAAATTTAAATACATCATAGCTCAGCTGATGAGG
Huh7D         AGGAAAGTGGAATCACGGATTAGCGACAAATTTAAATACATCATAGCTCAGCTGATGAGG
Huh7.5        AGGAAAGTGGAATCACGGATTAGCGACAAATTTAAATACATCATAGCTCAGCTGATGAGG
              1381.....1390......1400......1410......1420......1430.......
Huh7          GACACAGAGAGTCTGGCAAAGAGAATCTGCAAAGACCTCGAAAACTTATCTCAAATTCAA
Huh7D         GACACAGAGAGTCTGGCAAAGAGAATCTGCAAAGACCTCGAAAACTTATCTCAAATTCAA
Huh7.5        GACACAGAGAGTCTGGCAAAGAGAATCTGCAAAGACCTCGAAAACTTATCTCAAATTCAA
              1441.....1450......1460......1470......1480......1490.......
Huh7          AATAGGGAATTTGGAACACAGAAATATGAACAATGGATTGTTACAGTTCAGAAAGCATGC
Huh7D         AATAGGGAATTTGGAACACAGAAATATGAACAATGGATTGTTACAGTTCAGAAAGCATGC
Huh7.5        AATAGGGAATTTGGAACACAGAAATATGAACAATGGATTGTTACAGTTCAGAAAGCATGC
              1501.....1510......1520......1530......1540......1550.......
Huh7          ATGGTGTTCCAGATGCCAGACAAAGATGAAGAGAGCAGGATTTGTAAAGCCCTGTTTTTA
Huh7D         ATGGTGTTCCAGATGCCAGACAAAGATGAAGAGAGCAGGATTTGTAAAGCCCTGTTTTTA
Huh7.5        ATGGTGTTCCAGATGCCAGACAAAGATGAAGAGAGCAGGATTTGTAAAGCCCTGTTTTTA
              1561.....1570......1580......1590......1600......1610.......
Huh7          TACACTTCACATTTGCGGAAATATAATGATGCCCTCATTATCAGTGAGCATGCACGAATG
Huh7D         TACACTTCACATTTGCGGAAATATAATGATGCCCTCATTATCAGTGAGCATGCACGAATG
Huh7.5        TACACTTCACATTTGCGGAAATATAATGATGCCCTCATTATCAGTGAGCATGCACGAATG
              1621.....1630......1640......1650......1660......1670.......
```

```
Huh7        AAAGATGCTCTGGATTACTTGAAAGACTTCTTCAGCAATGTCCGAGCAGCAGGATTCGAT
Huh7D       AAAGATGCTCTGGATTACTTGAAAGACTTCTTCAGCAATGTCCGAGCAGCAGGATTCGAT
Huh7.5      AAAGATGCTCTGGATTACTTGAAAGACTTCTTCAGCAATGTCCGAGCAGCAGGATTCGAT
            1681.....1690......1700......1710......1720......1730.......
Huh7        GAGATTGAGCAAGATCTTACTCAGAGATTTGAAGAAAAGCTGCAGGAACTAGAAAGTGTT
Huh7D       GAGATTGAGCAAGATCTTACTCAGAGATTTGAAGAAAAGCTGCAGGAACTAGAAAGTGTT
Huh7.5      GAGATTGAGCAAGATCTTACTCAGAGATTTGAAGAAAAGCTGCAGGAACTAGAAAGTGTT
            1741.....1750......1760......1770......1780......1790.......
Huh7        TCCAGGGATCCCAGCAATGAGAATCCTAAACTTGAAGACCTCTGCTTCATCTTACAAGAA
Huh7D       TCCAGGGATCCCAGCAATGAGAATCCTAAACTTGAAGACCTCTGCTTCATCTTACAAGAA
Huh7.5      TCCAGGGATCCCAGCAATGAGAATCCTAAACTTGAAGACCTCTGCTTCATCTTACAAGAA
            1801.....1810......1820......1830......1840......1850.......
Huh7        GAGTACCACTTAAACCCAGAGACAATAACAATTCTCTTTGTGAAAACCAGAGCACTTGTG
Huh7D       GAGTACCACTTAAACCCAGAGACAATAACAATTCTCTTTGTGAAAACCAGAGCACTTGTG
Huh7.5      GAGTACCACTTAAACCCAGAGACAATAACAATTCTCTTTGTGAAAACCAGAGCACTTGTG
            1861.....1870......1880......1890......1900......1910.......

Huh7        GACGCTTTAAAAAATTGGATTGAAGGAAATCCTAAACTCAGTTTTCTAAAACCTGGCATA
Huh7D       GACGCTTTAAAAAATTGGATTGAAGGAAATCCTAAACTCAGTTTTCTAAAACCTGGCATA
Huh7.5      GACGCTTTAAAAAATTGGATTGAAGGAAATCCTAAACTCAGTTTTCTAAAACCTGGCATA
            1921.....1930......1940......1950......1960......1970.......
Huh7        TTGACTGGACGTGGCAAAACAAATCAGAACACAGGAATGACCCTCCCGGCACAGAAGTGT
Huh7D       TTGACTGGACGTGGCAAAACAAATCAGAACACAGGAATGACCCTCCCGGCACAGAAGTGT
Huh7.5      TTGACTGGACGTGGCAAAACAAATCAGAACACAGGAATGACCCTCCCGGCACAGAAGTGT
            1981.....1990......2000......2010......2020......2030.......
Huh7        ATATTGGATGCATTCAAAGCCAGTGGAGATCACAATATTCTGATTGCCACCTCAGTTGCT
Huh7D       ATATTGGATGCATTCAAAGCCAGTGGAGATCACAATATTCTGATTGCCACCTCAGTTGCT
Huh7.5      ATATTGGATGCATTCAAAGCCAGTGGAGATCACAATATTCTGATTGCCACCTCAGTTGCT
            2041.....2050......2060......2070......2080......2090.......
Huh7        GATGAAGGCATTGACATTGCACAGTGCAATCTTGTCATCCTTTATGAGTATGTGGGCAAT
Huh7D       GATGAAGGCATTGACATTGCACAGTGCAATCTTGTCATCCTTTATGAGTATGTGGGCAAT
Huh7.5      GATGAAGGCATTGACATTGCACAGTGCAATCTTGTCATCCTTTATGAGTATGTGGGCAAT
            2101.....2110......2120......2130......2140......2150.......
Huh7        GTCATCAAAATGATCCAAACCAGAGGCAGAGGAAGAGCAAGAGGTAGCAAGTGCTTCCTT
Huh7D       GTCATCAAAATGATCCAAACCAGAGGCAGAGGAAGAGCAAGAGGTAGCAAGTGCTTCCTT
Huh7.5      GTCATCAAAATGATCCAAACCAGAGGCAGAGGAAGAGCAAGAGGTAGCAAGTGCTTCCTT
            2161.....2170......2180......2190......2200......2210.......
Huh7        CTGACTAGTAATGCTGGTGTAATTGAAAAAGAACAAATAAACATGTACAAAGAAAAAATG
Huh7D       CTGACTAGTAATGCTGGTGTAATTGAAAAAGAACAAATAAACATGTACAAAGAAAAAATG
Huh7.5      CTGACTAGTAATGCTGGTGTAATTGAAAAAGAACAAATAAACATGTACAAAGAAAAAATG
            2221.....2230......2240......2250......2260......2270.......
Huh7        ATGAATGACTCTATTTTACGCCTTCAGACATGGGACGAAGCAGTATTTAGGGAAAAGATT
Huh7D       ATGAATGACTCTATTTTACGCCTTCAGACATGGGACGAAGCAGTATTTAGGGAAAAGATT
Huh7.5      ATGAATGACTCTATTTTACGCCTTCAGACATGGGACGAAGCAGTATTTAGGGAAAAGATT
            2281.....2290......2300......2310......2320......2330.......
Huh7        CTGCATATACAGACTCATGAAAAATTCATCAGAGATAGTCAAGAAAAACCAAAACCTGTC
Huh7D       CTGCATATACAGACTCATGAAAAATTCATCAGAGATAGTCAAGAAAAACCAAAACCTGTC
Huh7.5      CTGCATATACAGACTCATGAAAAATTCATCAGAGATAGTCAAGAAAAACCAAAACCTGTC
            2341.....2350......2360......2370......2380......2390.......
Huh7        CCTGATAAGGAAAATAAAAAACTGCTCTGCAGAAAGTGCAAAGCCTTGGCATGTTACACA
Huh7D       CCTGATAAGGAAAATAAAAAACTGCTCTGCAGAAAGTGCAAAGCCTTGGCATGTTACACA
Huh7.5      CCTGATAAGGAAAATAAAAAACTGCTCTGCAGAAAGTGCAAAGCCTTGGCATGTTACACA
            2401.....2410......2420......2430......2440......2450.......
Huh7        GCTGACGTAAGAGTGATAGAGGAATGCCATTACACTGTGCTTGGAGATGCTTTTAAGGAA
Huh7D       GCTGACGTAAGAGTGATAGAGGAATGCCATTACACTGTGCTTGGAGATGCTTTTAAGGAA
Huh7.5      GCTGACGTAAGAGTGATAGAGGAATGCCATTACACTGTGCTTGGAGATGCTTTTAAGGAA
            2461.....2470......2480......2490......2500......2510.......
```

```
Huh7      TGCTTTGTGAGTAGACCACATCCCAAGCCAAAGCAGTTTTCAAGTTTTGAAAAAAGAGCA
Huh7D     TGCTTTGTGAGTAGACCACATCCCAAGCCAAAGCAGTTTTCAAGTTTTGAAAAAAGAGCA
Huh7.5    TGCTTTGTGAGTAGACCACATCCCAAGCCAAAGCAGTTTTCAAGTTTTGAAAAAAGAGCA
          2521.....2530......2540......2550......2560......2570.......

Huh7      AAGATATTCTGTGCCCGACAGAACTGCAGCCATGACTGGGGAATCCATGTGAAGTACAAG
Huh7D     AAGATATTCTGTGCCCGACAGAACTGCAGCCATGACTGGGGAATCCATGTGAAGTACAAG
Huh7.5    AAGATATTCTGTGCCCGACAGAACTGCAGCCATGACTGGGGAATCCATGTGAAGTACAAG
          2581.....2590......2600......2610......2620......2630.......
Huh7      ACATTTGAGATTCCAGTTATAAAAATTGAAAGTTTTGTGGTGGAGGATATTGCAACTGGA
Huh7D     ACATTTGAGATTCCAGTTATAAAAATTGAAAGTTTTGTGGTGGAGGATATTGCAACTGGA
Huh7.5    ACATTTGAGATTCCAGTTATAAAAATTGAAAGTTTTGTGGTGGAGGATATTGCAACTGGA
          2641.....2650......2660......2670......2680......2690.......
Huh7      GTTCAGACACTGTACTCGAAGTGGAAGGACTTTCATTTTGAGAAGATACCATTTGATCCA
Huh7D     GTTCAGACACTGTACTCGAAGTGGAAGGACTTTCATTTTGAGAAGATACCATTTGATCCA
Huh7.5    GTTCAGACACTGTACTCGAAGTGGAAGGACTTTCATTTTGAGAAGATACCATTTGATCCA
          2701.....2710......2720......2730......2740......2750.......
Huh7      GCAGAAATGTCCAAATGA
Huh7D     GCAGAAATGTCCAAATGA
Huh7.5    GCAGAAATGTCCAAATGA
          2761.....2770.....
```
